# Near-Infrared Spectroscopy Demonstrates the Benefit of Erythracytapheresis in Sickle Cell Disease Adult Patients with Cerebral Vasculopathy

**DOI:** 10.3390/jcm12041256

**Published:** 2023-02-05

**Authors:** Suella Martino, Rym Chouk Turki, Fouzia Zouiti, Romain Fort, Sadaf Pakdaman, Stéphanie Forté, Dehbia Menouche, David Calvet, Thomas Rupp, France Pirenne, Pablo Bartolucci

**Affiliations:** 1 Sickle Cell Referral Center, Department of Internal Medicine, Henri Mondor University Hospital, UPEC, APHP, 94000 Creteil, France; 2 Etablissement Français du Sang, Île-de-France Mondor, 94000 Creteil, France; 3 Department of Internal Medicine, Edouard Herriot University Hospital, 69003 Lyon, France; 4 Division of Hematology and Oncology, Department of Medicine, Centre Hospitalier de l'Université de Montréal, Montreal, QC H2X 3E4, Canada; 5 Department of Apheresis, Henri Mondor University Hospital, UPEC, APHP, 94000 Creteil, France; 6 Department of Neurology, Sainte-Anne Hospital, 75014 Paris, France; 7 Interuniversity Laboratory of Human Movement Biology, University Savoie Mont Blanc, 73000 Chambery, France; 8 Laboratoire D’Excellence, GRex, Institut Mondor, INSERM U955 Equipe 2, 94000 Creteil, France

**Keywords:** sickle cell disease, NIRS, cerebral vasculopathy

## Abstract

Background: Cerebral vasculopathy can induce chronic cerebral hypoperfusion leading to stroke in patients with sickle cell disease (SCD) and is treated by blood exchange transfusion (BET). However, no prospective clinical study has demonstrated the benefit of BET in adults with SCD and cerebral vasculopathy. Near Infrared Spectroscopy (NIRS) is a recent non-invasive method complementary to Magnetic Resonance Imaging (MRI). We evaluated cerebral perfusion using NIRS during erythracytapheresis in patients with SCD with and without steno-occlusive arterial disease. Methods: We conducted a monocentric, prospective study in 16 adults with SCD undergoing erythracytapheresis in 2014. Among them, 10 had cerebral steno-occlusive arterial disease. NIRS measured the relative amounts of oxyhemoglobin (OxyHb), deoxyhemoglobin (DeoxyHb) and total hemoglobin (Total Hb) in brain tissue and in muscle. Results: In cerebral hemispheres associated with steno-occlusive arterial disease, we observed a significant increase of OxyHb and Total Hb during BET, without modification of DeoxyHb. Conclusion: Using NIRS during BET showed that BET improves cerebral perfusion in adult patients with SCD with cerebral vasculopathy.

## 1. Introduction

Sickle cell disease (SCD) is a severe monogenic disorder characterized by chronic hemolytic anemia and vascular dysfunction. 

Cerebral vasculopathy, usually appearing during childhood, can induce chronic cerebral hypoperfusion and progressive cerebral atrophy. This can lead to cognitive impairment and/or stroke with a cumulative incidence of 11% by age 20 in the United States Cooperative Study of SCD [1]. Prevention of these complications is necessary to reduce the incidence of disability and improve the quality of life of these patients. Screening for cerebral steno-occlusive arterial disease is done using transcranial Doppler (TCD) for the detection of accelerated velocities in children with SCD [2,3]. The cornerstone of cerebral vasculopathy treatment is blood exchange transfusion (BET) to lower the percentage of HbS ≤ 30%, mainly by erythracytapheresis [1,4,5]. However, no prospective clinical study has been conducted to demonstrate the benefits of BET in adults with cerebral vasculopathy. Moreover, BET was unable to prevent stroke in SS patients [6,7,8]. On the other hand, transfusions are associated with complications including delayed hemolysis transfusion reactions [9,10], infections and thrombosis on catheters [11] or arterio-veinous fistula [12], and represent a burden on blood delivery. It is therefore necessary to provide evidence of a beneficial effect of BET in adults with cerebral vasculopathy. 

Since brain vasculopathy is present, the goal of therapeutic management is principally to improve cerebral perfusion. Cerebral perfusion can be assessed by analyzing blood flow using Magnetic Resonance Imaging with a Time of Flight sequence (MRI), Single Photon Emission Computed Tomography (SPECT), Dynamic Susceptibility Contrast (DSC) MRI with Gadolinium injection, and Pseudo-Continuous Arterial Spin Labeling (PCASL) MRI.

Near Infrared Spectroscopy (NIRS) has emerged in recent years as a non-invasive method complementary to MRI to measure relative amounts of oxyhemoglobin (OxyHb), deoxyhemoglobin (DeoxyHb), and total hemoglobin (Total Hb) in brain tissue [13]. NIRS is an optical imaging technique based on the difference in transmitted and received photons at near-infrared wavelengths. Contrary to other methods, NIRS can be performed during BET for 2 h without adverse effect. The spatial resolution of NIRS is superior to TCD, which only detects total blood flow.

Because of the occurrence of stroke in the immediate aftermath of erythracytapheresis, we designed a study to evaluate cerebral perfusion using NIRS during erythracytapheresis in patients with SCD with and without steno-occlusive arterial disease.

## 2. Materials and Methods

### 2.1. Study Population

We conducted a monocentric, prospective study in adults with SCD homozygous hemoglobinopathy (SS) followed in our university hospital Henri Mondor and undergoing erythracytapheresis. 

This study was approved by our institutional review board in accordance with the Declaration of Helsinki and the French Data Protection Authority (“Commission Nationale Informatique et Liberté;s”, CNIL, authorization n°911539, and “Comité consultatif sur le traitement de l’information en matiére de recherche dans le domaine de la santé”, CCTIRS, authorization n°11.537, Paris, France). Written informed consent was obtained from all patients. 

Inclusion criteria were SS or Sβ_0_ thal patients older than 18 years of age, on chronic BET by erythracytapheresis and having had a brain MRI. Exclusion criteria were the following: patients refusing to sign the written consent, patients incapable of giving their consent, vaso-occlusive crisis requiring an emergency room visit or stroke during the 3 months preceding inclusion, pregnant or breast-feeding women, patients with no- or retained freedom. Vital signs and blood samples were obtained on the same day for each patient prior to and after transfusion. Complete blood count, p 50 levels by venous blood gas (Radiometer, ABL 800, Danemark) and quantitative hemoglobin (Hb) electrophoresis by high performance liquid chromatography (HPLC) (BioradTM, Variant II automaton, betathal dual, program, France) were analyzed in our clinical laboratory. Steno-occlusive vasculopathy was defined as a stenosis > 50% on an MRI [14]. We distinguished cerebral hemispheres with and without steno-occlusive arterial disease in each patient. 

### 2.2. Near-Infrared Spectroscopy (NIRS)

NIRS is an optical non-invasive imaging technology that measures perfusion modifications by indirectly assessing activity with light of a specific near-infrared wavelength. NIRS relies mainly on two characteristics of human tissue: the relative transparency of tissue to light in the NIRS range, and the oxygenation-dependent light absorbing characteristics of Hb. It enables the measurement of changes in OxyHb and Deo-xyHb, which reflect local brain activity with an excellent resolution of brain signals in real-time. NIRS (Portalite, ARTINIS, Pays-Bas) was used to measure relative changes of OxyHb and DeoxyHb concentration (in ∆μmol) in the prefrontal cortex (PFC). The NIRS system operated with three wavelengths of light (775, 810, and 850 nm). Total Hb was equivalent to the sum of OxyHb and DeoxyHb. A modified Beer–Lambert law was used to calculate the relative change in OxyHb, DeoxyHb, and Total Hb.

After installation of the patient, the detection optodes were placed on the prefrontal areas just above the eyebrows (cerebral oxygenation) and on the vastus lateralis of the right leg (muscular oxygenation) as a control. PFC and muscle probe holders were secured to the skin using double-sided adhesive tape to minimize any change in its relative position, and optodes were covered with sweatbands to shield from surrounding light. The optodes were kept in place during the BET. The recording was started after a 10 min stability period, and continued before, during and after the BET.

OxyHb, DeoxyHb, and total Hb were recorded at the two cerebral hemispheres and at the muscular level continuously throughout the entire erythracytapheresis procedure. The variation of these parameters was calculated using predefined time points corresponding to: 1/ 10 min after stabilization of the curves and before the procedure, 2/ at the end of the procedure. Cerebral data were interpreted by hemisphere, differentiating between hemispheres with and without steno-occlusive arterial disease. Improvement in cerebral perfusion was defined by an increase in OxyHb and Total Hb between the two points, before and at the end of the erythracytapheresis. 

### 2.3. Statistical analyses

Statistical analyses were performed using GraphPad Prism (version 7.0; GraphPad Software). Qualitative data were expressed as percentages and were analyzed using the Pearson’s chi-squared test of independence. Quantitative data were expressed as medians (interquartile ranges, IQRs). The Wilcoxon test (paired), the Friedman test (paired) and the Mann–Whitney test (unpaired) were used as appropriate. *p*-values < 0.05 were considered statistically significant. 

## 3. Results

### 3.1. Demographics and NIRS Dynamics

#### 3.1.1. Demographics

Table 1 summarizes the demographic and biological data of the two participating groups. The groups were similar for median age, gender distribution, Hydroxyurea (HU) treatment and stroke. 

Sixteen adult patients with SCD on erythracytapheresis were enrolled from July 2014 to October 2014. Ten women and six men were included with an average age of 32 ± 9. Among them, 10 had cerebral steno-occlusive arterial disease accounting for 13 hemispheres associated with a vasculopathy (two left unilateral, five right unilateral, and three bilateral). The main indications for BET included cerebral steno-occlusive arterial disease with at least one episode of stroke (*n* = 7), cerebral steno-occlusive arterial disease without stroke (*n* = 3), autoimmune hepatitis (*n* = 1), vaso-occlusive crisis, priapism (*n* = 2), and three patients for which information was not available. The only stroke in the group without cerebral steno-occlusive arterial disease occurred in the context of meningitis in childhood. At BET onset, eight patients were treated by HU, of which six had cerebral steno-occlusive arterial disease, and two did not have cerebral steno-occlusive arterial di-sease.

Hb, hematocrit (Ht) and p50 were similar in the two groups before BET. Hemoglobin S (HbS) levels pre transfusion were significantly lower in patients with steno-occlusive arterial disease.

NIRS was analyzed for all subject groups. No complications were encountered during the measurement.

**Figure A1:**
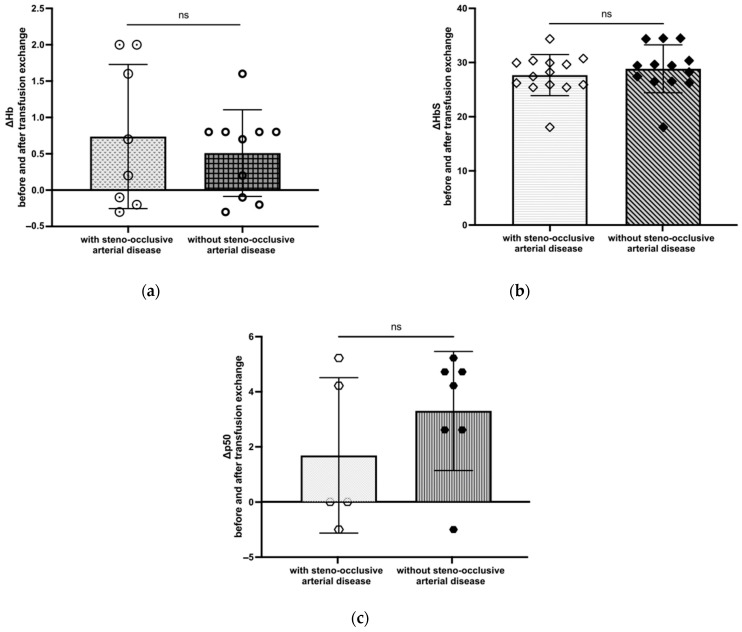
Measurement of ΔHb, ΔHbS, and Δp50 before and after BET in cerebral hemispheres with steno-occlusive arterial disease and without steno-occlusive arterial disease. Shaded boxes show values obtained. (**a**) ΔHb in cerebral hemispheres with steno-occlusive arterial disease (*n* = 8) and in cerebral hemispheres without steno-occlusive arterial disease (*n* = 10). ns using the Mann–Whitney test. (**b**) ΔHbS in cerebral hemispheres with steno-occlusive arterial disease (*n* = 14) and in cerebral hemispheres without steno-occlusive arterial disease (*n* = 13). ns using the Mann–Whitney test. (**c**) Δp50 in cerebral hemispheres with steno-occlusive arterial disease (*n* = 5) and in cerebral hemispheres without steno-occlusive arterial disease (*n* = 7). ns using the Mann–Whitney test.

Table 2 summarizes biological data for the 32 cerebral hemispheres of patients with SCD according to their cerebral steno-occlusive arterial disease status. Hb and Ht were similar in the two groups before and after BET. As expected, HbS levels post transfusion were significantly lower in both groups. p50 was evaluated only in 10 cerebral hemispheres with steno-occlusive arterial disease and in 8 without steno-occlusive arterial disease before BET (Appendix A Table A1). For cerebral hemispheres without steno-occlusive arterial disease, p50 had an abnormally higher level before BET, then p50 levels decreased significantly after BET (*p* = 0.03). Delta (Δ) is defined by subtracting the two values to determine the difference between two phases, before and after BET. In cerebral hemispheres with and without steno-occlusive arterial disease, we did not observe any difference regarding ΔHb, ΔHbS, and Δp50 (respectively, Appendix A Figure A1a–c). 

#### 3.1.2. NIRS dynamics

**Figure 1:**
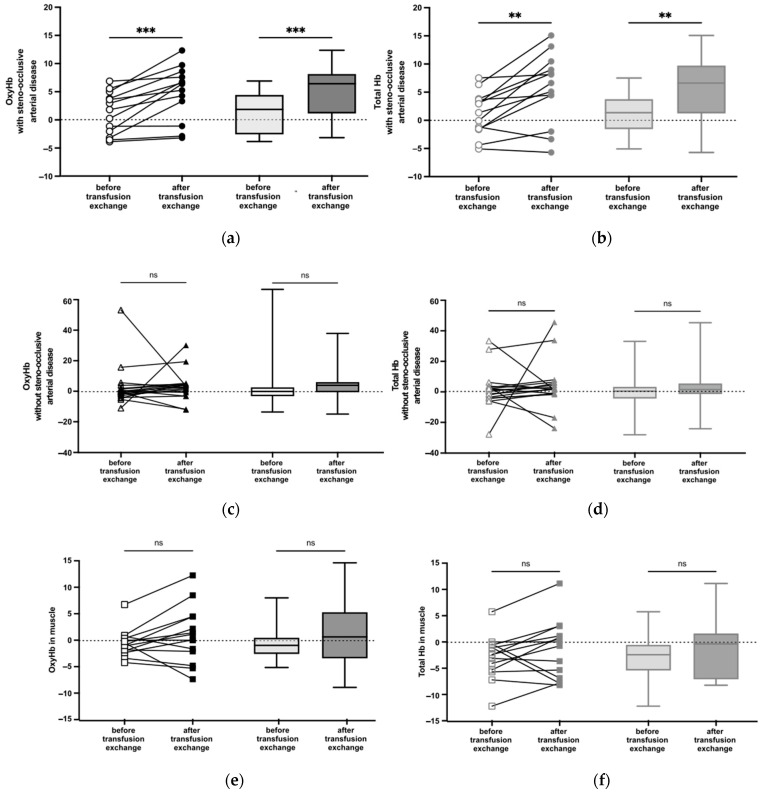
NIRS measurement of relative amounts of OxyHb and Total Hb before and after BET. Box whisker plots show range, interquartile percentages, and median values for OxyHb and Total Hb. (**a**) OxyHb in cerebral hemispheres with steno-occlusive arterial disease (*n* = 13). ***, *p* < 0.001 using the Wilcoxon test. (**b**) Total Hb in cerebral hemispheres with steno-occlusive arterial disease (*n* = 13). **, *p* < 0.01 using the Wilcoxon test. (**c**) OxyHb in cerebral hemispheres without steno-occlusive arterial disease (*n* = 19). ns, not significant using the Wilcoxon test. (**d**) Total Hb in cerebral hemispheres without steno-occlusive arterial disease (*n* = 18). ns using the Wilcoxon test. (**e**) OxyHb in muscle (*n* = 14). ns using the Wilcoxon test. (**f**) Total Hb in muscle (*n* = 14). ns using the Wilcoxon test.

**Figure A2:**
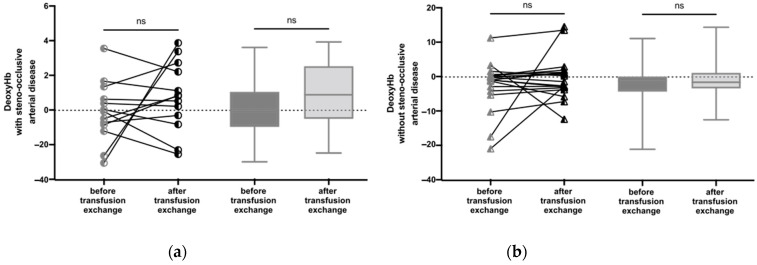

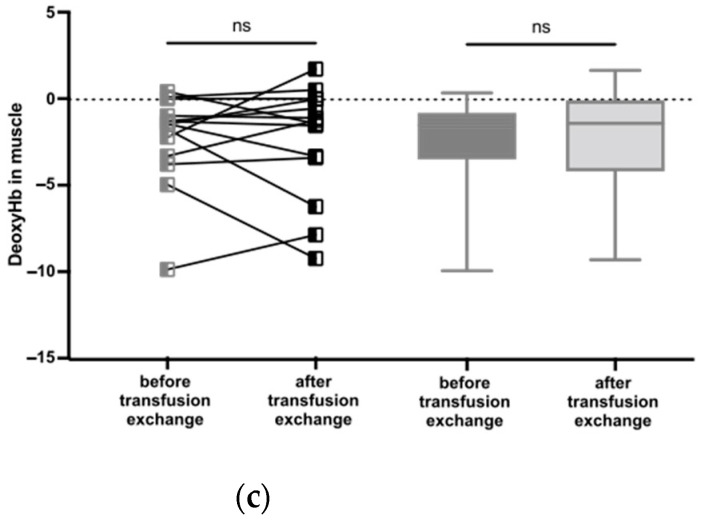
Measurement of the relative amounts of DeoxyHb before and after BET. Box whisker plots show range, interquartile percentages, and median values for DeoxyHb. (**a**) DeoxyHb in cerebral hemispheres with steno-occlusive arterial disease (*n* = 13). ns using the Wilcoxon test. (**b**) DeoxyHb in cerebral hemispheres without steno-occlusive arterial disease (*n* = 19). ns using the Wilcoxon test. (**c**) DeoxyHb in muscle (*n* = 14). ns using the Wilcoxon test.

In cerebral hemispheres associated with steno-occlusive arterial disease, we observed a significant increase of OxyHb (*p* = 0.0002) (Figure 1a) and Total Hb (*p* = 0.003) (Figure 1b) during BET, without modification of DeoxyHb (Appendix A Figure A2a). In cerebral hemispheres not associated with steno-occlusive arterial disease, as well as in muscle, we did not observe any difference regarding OxyHb and Total Hb after BET (Figure 1c–f, respectively).

**Figure 2:**
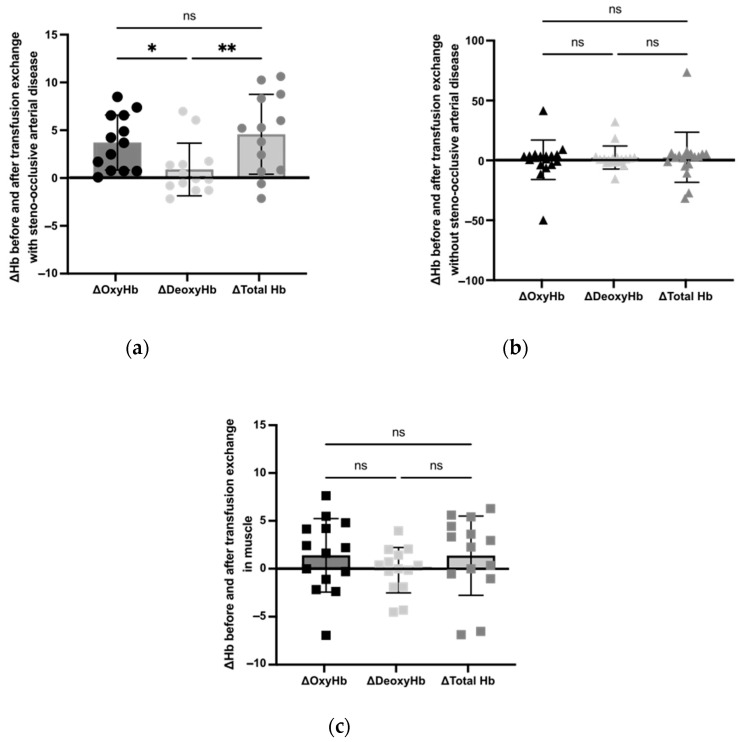
Measurement of ΔOxyHb, ΔDeoxyHb, and ΔTotal Hb before and after BET. Shaded boxes show values obtained. (**a**) ΔOxyHb, ΔDeoxyHb, and ΔTotal Hb in cerebral hemispheres with steno-occlusive arterial disease (*n* = 13). *, *p* < 0.05; **, *p* < 0.01 using the Friedman test. (**b**) ΔOxyHb, ΔDeoxyHb, and ΔTotal Hb in cerebral hemispheres without steno-occlusive arterial disease (*n* = 18). ns using the Friedman test. (**c**) ΔOxyHb, ΔDeoxyHb, and ΔTotal Hb in muscle (*n* = 14). ns using the Friedman test.

The increase in ΔOxyHb (*p* = 0.018) and ΔTotal Hb (*p* = 0.005) seen in hemispheres with steno-occlusive arterial disease was significantly higher than for ΔDeoxyHb (Figure 2a). 

We did not observe any difference regarding ΔOxyHb, ΔTotal Hb and ΔDeoxyHb in hemispheres not associated with vasculopathy and in muscle (respectively, Figure 2b,c).

## 4. Discussion

Our paper presents real-time evidence of cerebral perfusion improvement during erythracytapheresis in adult patients with SCD with cerebral vasculopathy, as shown by the significant increase of OxyHb and Total Hb only in hemispheres with steno-occlusive arterial disease. Furthermore, the increase in ΔTotal Hb was mainly related to an improvement in the oxygenated arterial blood supply, as shown by the increase in ΔOxyHb with no change in ΔDeoxyHb. The lack of increase in ΔTotal Hb in hemispheres not associated with vasculopathy and in muscle suggests that the modification of hemoglobin’s oxygen affinity due to the replacement of the patients’ red blood cells (RBC) with AA donor RBCs is not responsible for the increase in ΔOxyHb. 

**Figure A3:**
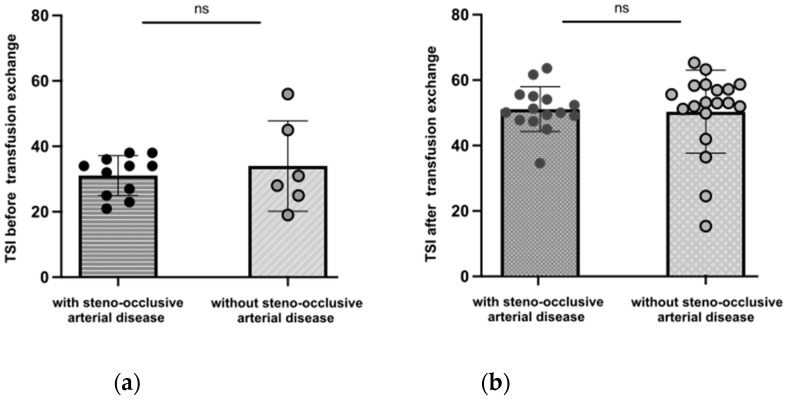
NIRS measurement of TSI (%) in cerebral hemispheres with and without steno-occlusive arterial disease before and after BET. Box whisker plots show range, interquartile percentages for TSI. (a) TSI before transfusion (with steno-occlusive arterial disease, *n* = 15 and without steno-occlusive arterial disease, *n* = 19). (b) TSI after transfusion (with steno-occlusive arterial disease, *n* = 15 and without steno-occlusive arterial disease, *n* = 19). ns using the Mann–Whitney test.

Measures of OxyHb, DeoxyHb and Total Hb were highly specific. Surprisingly, we observed no difference in peripheral capillary oxygen saturations using the Tissue Saturation Index (TSI) by NIRS in the two groups before and after transfusion (Appendix A Figure A3).

Until now, adult recommendations for cerebral vasculopathy were based on pediatric studies clearly demonstrating a reduction in the risk of stroke under a BET program with a target of 30% in HbS (STOP1 and 2 studies) [3,15]. In these studies, the risk of having a stroke was high in children with TCD accelerations greater than 200 cm/s. The 92% risk reduction with transfusion programs was associated with a reduction in TCD velocities, despite the fact that all children with abnormal TCD did not necessarily have a stenosis visible on the MRI. 

Adult cerebral vasculopathy differs from children’s because the acceleration of velocities is always related to a stenosis visible on the MRI [16]. The situation is therefore equivalent to that of children with stenosis detected with TCD, but which is also visible on MRI. Moreover, the risk of having stroke recurrence in adulthood is higher than during childhood, despite similar treatments [17]. We observed a significantly lower HbS levels in patients with steno-occlusive arterial disease before transfusion. This was not surprising since the therapeutic goal is to have an HbS level < 30% in patients with steno-occlusive indications, whereas patients without steno-occlusive arterial disease have higher HbS targets according to French guidelines [18]. 

In the case of established vasculopathy, the risk of having a stroke is directly dependent on cerebral perfusion. The latter is dependent on many parameters besides the stenosis itself, such as cerebral perfusion pressure, Hb concentration, partial arterial carbon dioxide pressure (PaCO2), partial arterial pressure of oxygen (PaO2), glycemia, temperature, jugular venous return, or intracranial pressure. Furthermore, the significant hemorrheological alterations, in particular of viscosity [19] and oxygen transport [20], that are found in SCD contribute qualitatively to these perfusion abnormalities.

Given the complexity of cerebral perfusion in these patients, as well as the incidence of stroke immediately following erythracytapheresis, confirming the benefit of this procedure in improving cerebral perfusion in patients with cerebral vasculopathy, and therefore at risk of stroke recurrence, was necessary. One of our fears was indeed that BET would increase perfusion of healthy areas to the detriment of poorly perfused areas (vascular theft). 

NIRS allows for the monitoring of hemoglobin in its oxy- and deoxygenated forms during erythracytapheresis, in a cerebral volume corresponding to the most affected brain areas. Patients with cerebral vasculopathy mainly have anterior border zone lesions between the territory of the anterior cerebral and the middle cerebral arteries [6]. To verify that Hb modifications were not only linked to a change in the quality of blood due to erythracytapheresis, but also associated with improvement in cerebral perfusion, we performed control NIRS of the same parameters in the same individual, but at the muscular level. This area is widely described with this technique during physical activity. Our study shows an increase in total Hb in the affected areas, indicating an improvement in cerebral perfusion. This was not the case in the muscle or in hemispheres without vascular damage. The increase in total Hb was mainly related to an increase in the oxygenated arterial blood supply, as shown by the increase in OxyHb with no change in DeoxyHb. Cerebral perfusion improvement could be due to a hemorheological improvement, such as a rapid drop in viscosity [21], having a greater beneficial effect in the vascular areas affected. It may also be related to the use of the cerebrovascular reserve (CVR), which does not exist in the muscle. The vaso-reactivity that underlies the triggering of CVR is modulated by changes in acidity or PaCO2, which are used during scintigraphy or MRI with acetazolamide, or during respiratory maneuvers. However, our study cannot determine whether the improvements observed are related to these modifications and whether the perfusion at erythracytapheresis initiation was normal or not. 

Despite our encouraging results, NIRS has some limitations [22]. NIRS is a relative quantification and not an absolute one, with necessary real-time monitoring. The performance of these examinations by two trained physicians according to a strict procedure with predefined temporal analysis criteria makes it possible to reduce reproducibility bias. Nevertheless, we did not evaluate the variability of NIRS results within the same patient using repeated measures. Furthermore, NIRS receives information not only from the cerebral cortex, but also from skin and bones, which increases the complexity of information extraction. For instance, a thick cranial vault or a significant cerebral atrophy can distort the results. Even if the analysis of the spectra targets Hb over a depth of 2 cm of tissue, we cannot exclude biases in the analysis of the cranial vault. However, the absence of improvement in the unaffected cerebral hemispheres does not support this hypothesis. Finally, the small number of patients is also a limitation of this study. 

Surprisingly, p50 had an abnormal lower level in patients with cerebral steno-occlusive arterial disease before and after transfusion. In fact, studies report that p50 is 27 mmHg in individuals with HbA, and 30.3 mmHg in SS patients [23]. In addition, oxygen dissociation curves of patients with SCD tend to be shifted to the right, whereas transfused RBC are shifted to the left due to 2,3-diphosphoglyceric acid (2,3-DPG) degradation during storage [24]. Our study shows an improvement in cerebral perfusion after BET in SCD hemispheres with steno-occlusive arterial disease. Thus, this conflicting evidence may suggest that other mechanisms independent of p50 allow for enhanced cerebral oxygen extraction [25]. 

Finally, we attributed the stroke occurring immediately after erythracytapheresis to a vasovagal syncope. These are not rare after the procedure and particular caution must be taken to avoid them as much as possible, as their consequences can be disastrous in patients at risk of cerebral hypoperfusion. 

In conclusion, our study shows that cerebral perfusion was significantly improved by erythracytapheresis in cerebral hemispheres with steno-occlusive arterial disease in adult patients with SCD. These results also confirm the feasibility of evaluating real-time cerebral perfusion with NIRS. 

## Figures and Tables

**Table 1:** Main clinical and biological variables from patients with SCD in two subgroups according to their cerebral steno-occlusive arterial disease status.

Characteristics	Total (*n* = 16)	With Steno-Occlusive (*n* = 10)	Without Steno-Occlusive (*n* = 6)	*p*-Value
Median age, years	31.5 [25–36.5]	33 [25.5–35.5]	29.5 [25.8–41.5]	0.98
Female gender, *n* (%)	10 (62.5)	7 (70)	3 (50)	0.42
Hydroxyurea, *n* (%)	8 (50)	6 (60)	2 (50)	0.45
Stroke, *n* (%)	8 (50)	7 (70)	1 (16.7)	0.02
Hemoglobin, g/dL	10 [8.7–10.2]	9.8 [8.4–10.2] (*n* = 10)	9.1 [8.75–9.75] (*n* = 3)	0.96
Hematocrit, %	28 [24–30]	28 [24–30] (*n* = 10)	26 [25–29] (*n* = 3)	0.85
Hemoglobin S, g/dL	41.5 [38.6–49.1]	40.65 [38.2–44.7] (*n* = 10)	53 [51.1–59.55] (*n* = 3)	0.007
p50	27.2 [26.8–29.4]	26.8 [26.3–27.45] (*n* = 7)	30.45 [29.9–31] (*n* = 2)	0.25

**Table 2:** Main biological variables from patients with SCD according to their cerebral steno-occlusive arterial disease status. ^†^ Missing data (MD).

Characteristics		Pre Transfusion		Post Transfusion	*p*-Value
with steno-occlusive (*n* = 13)	MD ^†^		MD ^†^		
Hemoglobin, g/dL	0	9.6 [8.1–10.2]	5	10.1 [9.6–10.1]	0.172
Hematocrit, %	0	28 [24–30]	5	30 [27.8–30]	0.188
Hemoglobin S, g/dL	0	39.7 [37.8–43.8]	0	13.5 [10.6–14.4]	0.0002
p50	3	27 [26.8–27.7]	8	26.8 [22–26.8]	0.5
without steno-occlusive (*n* = 19)	MD ^†^		MD ^†^		
Hemoglobin, g/dL	6	10 [9.1–10.2]	7	9.9 [9.6–10.1]	0.043
Hematocrit, %	6	28 [26–30]	7	28 [27.8–30]	0.19
Hemoglobin S, g/dL	6	49.2 [45.1–53]	6	18.7 [17.8–19.9]	0.0002
p50	11	29.4 [29.4–31.5]	12	26.8 [26.8–26.8]	0.03

## Data Availability

The datasets will be available from the corresponding author on reasonable request.

## Appendix A

**Table A1:** Main biological variables from patients with SCD according to their cerebral steno-occlusive arterial disease status. Quantitative data were expressed as medians [interquartile ranges, IQRs]. The nonparametric Wilcoxon test was used as appropriate. *p*-values < 0.05 were considered statistically significant. ^†^ Missing data (MD).

Characteristics		Pre Transfusion		Post Transfusion	*p*-Value
with steno-occlusive (*n* = 13)	MD ^†^		MD ^†^		
p50	3	27 [26.8-27.7]	8	26.8 [22-26.8]	0.25
without steno-occlusive (*n* = 19)					
p50	11	29.4 [29.4-31.5]	12	26.8 [26.8-26.8]	0.03
